# Invasive fractional-flow-reserve prediction by coronary CT angiography using artificial intelligence vs. computational fluid dynamics software in intermediate-grade stenosis

**DOI:** 10.1007/s10554-024-03173-0

**Published:** 2024-07-04

**Authors:** Benjamin Peters, Jean-François Paul, Rolf Symons, Wouter M.A. Franssen, Alain Nchimi, Olivier Ghekiere

**Affiliations:** 1 Faculty of Medicine and Life Sciences, Hasselt University, LCRC, Agoralaan, Diepenbeek, 3590 Belgium; 2 Department of Radiology, Jessa Hospital, LCRC, Stadsomvaart 11, Hasselt, 3500 Belgium; 3 Department of Radiology, Institut Mutualiste Montsouris, 42 Boulevard Jourdan, Paris, France; 4 Department of Radiology, Imelda Hospital, Bonheiden, Belgium; 5 SMRC Sports Medical Research Center, BIOMED Biomedical Research Institute, Faculty of Medicine and Life Sciences, Hasselt University, Diepenbeek, Belgium; 6 GIGA Cardiovascular Sciences, Liège University (ULg), Domaine Universitaire du Sart Tilman, rue de l’Hôpital, Liège, Belgium; 7 Department of Radiology, Centre Hospitalier Universitaire, Luxembourg, Luxembourg, Luxembourg

**Keywords:** Fractional flow reserve, Coronary computed tomography angiography, Artificial intelligence, Deep learning, Intermediate coronary stenoses

## Abstract

Coronary computed angiography (CCTA) with non-invasive fractional flow reserve (FFR) calculates lesion-specific ischemia when compared with invasive FFR and can be considered for patients with stable chest pain and intermediate-grade stenoses according to recent guidelines. The objective of this study was to compare a new CCTA-based artificial-intelligence deep-learning model for FFR prediction (FFR_AI_) to computational fluid dynamics CT-derived FFR (FFR_CT_) in patients with intermediate-grade coronary stenoses with FFR as reference standard. The FFR_AI_ model was trained with curved multiplanar-reconstruction CCTA images of 500 stenotic vessels in 413 patients, using FFR measurements as the ground truth. We included 37 patients with 39 intermediate-grade stenoses on CCTA and invasive coronary angiography, and with FFR_CT_ and FFR measurements in this retrospective proof of concept study. FFR_AI_ was compared with FFR_CT_ regarding the sensitivity, specificity, positive predictive value (PPV), negative predictive value (NPV), and diagnostic accuracy for predicting FFR ≤ 0.80. Sensitivity, specificity, PPV, NPV, and diagnostic accuracy of FFR_AI_ in predicting FFR ≤ 0.80 were 91% (10/11), 82% (23/28), 67% (10/15), 96% (23/24), and 85% (33/39), respectively. Corresponding values for FFR_CT_ were 82% (9/11), 75% (21/28), 56% (9/16), 91% (21/23), and 77% (30/39), respectively. Diagnostic accuracy did not differ significantly between FFR_AI_ and FFR_CT_ (*p* = 0.12). FFR_AI_ performed similarly to FFR_CT_ for predicting intermediate-grade coronary stenoses with FFR ≤ 0.80. These findings suggest FFR_AI_ as a potential non-invasive imaging tool for guiding therapeutic management in these stenoses.

## Introduction

Coronary computed tomography angiography (CCTA) is the first-line radiological investigation for patients with stable chest pain, according to guidelines issued by the European Society of Cardiology in 2019, [1] the American Heart Association (AHA) and American College of Cardiology in 2022. [2] However, coronary stenosis morphology correlates poorly with functional impact, notably in the intermediate-grade range. [3] Consequently, fractional flow reserve (FFR) measurement during invasive coronary angiography (ICA) is recommended in 2022 guidelines on coronary artery revascularization. [4] However, invasive FFR measurement is not widely performed in clinical practice given the increased risk of ICA complications and additional cost. [4] According to recent guidelines, computational fluid dynamics (CFD) based FFR calculations on CCTA (FFR_CT_)(HeartFlow Analysis, HeartFlow, Mountain View, CA) can be considered for patients with stable, recent-onset chest pain and intermediate grade stenosis. [2]

In contrast to CFD models, CorEx (Spimed-AI, Paris, France) is a new semi-automated program which uses a supervised artificial-intelligence (AI) CCTA-based deep-learning model (DLM) to predict FFR. This model has already been validated for CAD-RADS classification. [5] Several other AI DLMs have been designed for FFR prediction showing variable results (diagnostic accuracies between 66% and 79%), including a wide range of stenoses and without comparison with FFR_CT_. [6, 7] Therefore, the objective of this retrospective study was to compare a new FFR_AI_ model to FFR_CT_ for predicting FFR ≤ 0.80 in intermediate-grade stenoses.

## Methods

### Patients and study design

The study protocol was approved by our institutional review board. In compliance with Belgian law on retrospective analyses of de-identified health data, patient informed consent was not required.

We identified the study participants retrospectively by searching our institutional database for patients with intermediate-grade coronary stenoses assessed by both CCTA and ICA as previously reported. [8] The study population was not part of the training set to create FFR_AI_. The patients were included in a previous study designed to compare FFR_CT_ and cardiac magnetic resonance imaging FFR estimates to FFR values. [8] Intermediate-grade stenoses were defined as 40–70% diameter reduction on quantitative coronary angiography. [4] CCTA, FFR_CT_ computation, and ICA with pressure wire-derived FFR measurements were performed as previously described. [8] The same CCTA data were used to determine FFR_AI_ and compared to the FFR values obtained during ICA.

### Deep-learning FFR_AI_ model

To develop the CorEx AI model (Spimed-AI), we designed a DLM architecture derived from the convolutional neural network (CNN) InceptionV3 (Google, Mountain View, CA). The model has two components, one specifically designed to extract relevant features from images and the other to classify the extracted features. This second component is composed of symmetric and asymmetric building blocks containing convolutions followed by maximum-pooling and concatenation.

To maximize performance, the model was fed with two inputs: the nine cMPR image and a vector of size 99 composed of the output of 11 other classifications from each of the nine cMPRs. The final output uses a softmax activation with a binary FFR classification as ≤ 0.80 or > 0.80, as recommended in the 2018 ESC/EACTS and 2021 ACC/AHA/SCAI Guidelines on myocardial revascularization. [4, 9]

For training, the model weights were initialized randomly according to the normal distribution, and biases were zero-initialized. Various random data augmentations were performed on the input data, such as vertical flip, horizontal flip, translations, scaling, rotation, additive Gaussian noise, additive Laplace noise, additive Poisson noise, brightness shift, and motion blur simulation. For each training sequence, only those weights maximizing the validation Matthews correlation coefficient (MCC) over all epochs were kept.

We created a training dataset comprised of 4500 curved cMPR images from 500 stenotic vessels in 413 patients who had at least one coronary stenosis, with the corresponding FFR values. For each stenotic artery, cMPR images were extracted at 40° intervals over the 360° circumference around the coronary centerline, yielding nine images in all. The centerlines were corrected manually if necessary (notably for calcified arteries) by a cardiac radiologist with 25 years of experience (JFP). Patients with stents and/or coronary-artery bypass grafting were not included in the training set. CCTA images were acquired using several different CT machines including a dual-source machine (Flash Definition, Siemens Healthineers, Erlangen, Germany), a 64-slice machine (Discovery CT 750 HD, GE, Milwaukee, WI), and a 320-detector machine (Aquilion One Genesis Edition, Canon Medical, Tochigi, Japan). Each image was annotated and classified considering the whole artery based on whether FFR was ≤ 0.80 or > 0.80, with cutoffs according to recent guidelines. [4, 9] The FFR measurements were performed by four different cardiology teams. Of the 500 stenotic coronary arteries in the training set, 125 (25%) had FFR values ≤ 0.80.

### Image analysis

#### Integration of FFR, FFR_CT_, and FFR_AI_

**Fig. 1:**
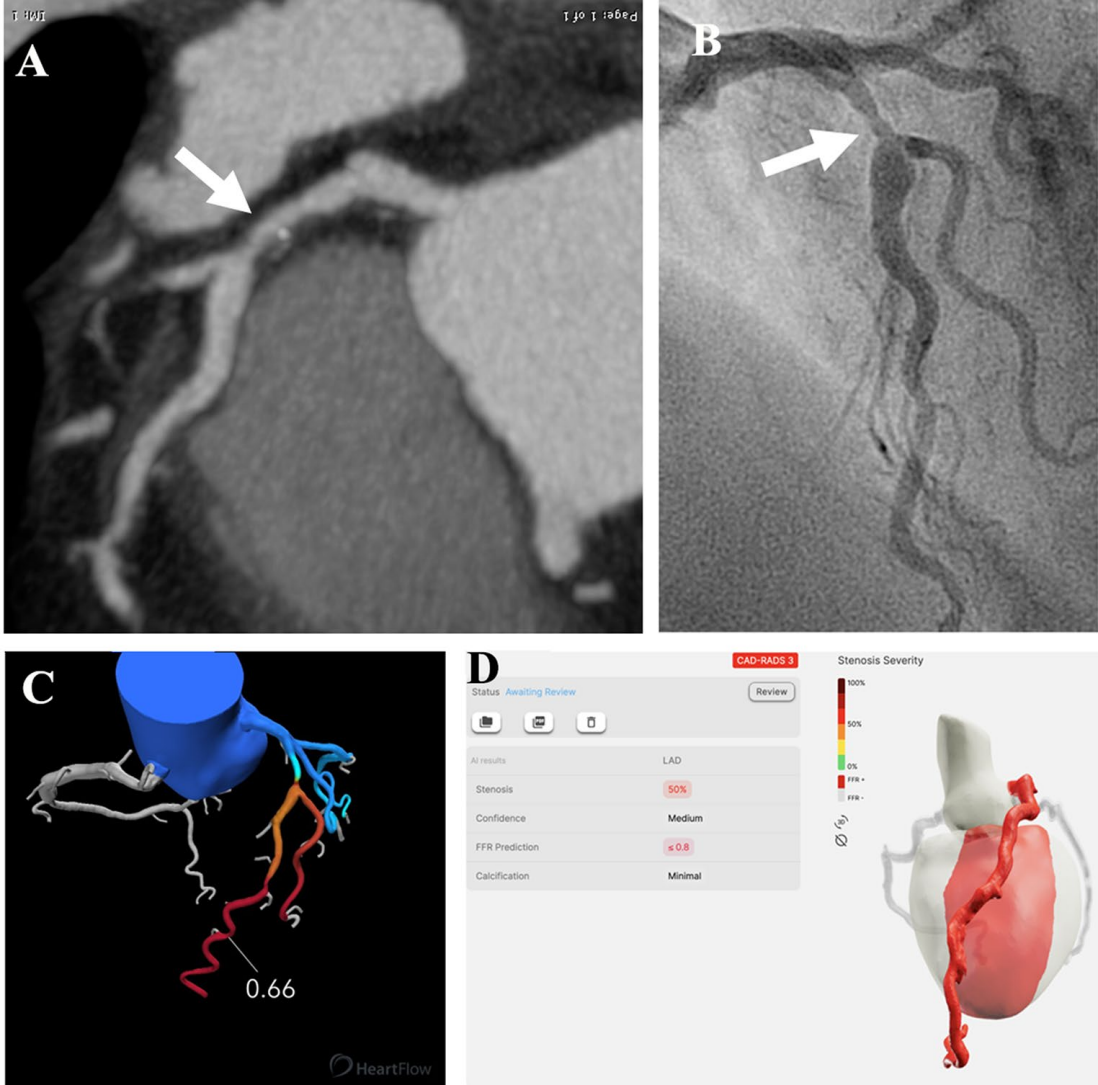
A 62-year-old patient presenting with stable chest pain. Curved multiplanar CCTA reconstructions of the left anterior descending artery show an intermediate-grade stenosis of the proximal segment (white arrow in **A**), confirmed by invasive coronary angiography (white arrow in **B**). Invasive FFR measurement was 0.61, FFR_CT_ analysis was 0.66 (**C**) and FFR_AI_ prediction was ≤ 0.80 (**D**). CCTA: Coronary computed tomography angiography; LAD: left anterior descending artery; FFR: Fractional flow reserve measured during invasive coronary angiography; FFR_CT_: fractional flow reserve (FFR) estimated by computational fluid dynamics software applied to CCTA images; FFR_AI_: FFR estimated by a deep learning model applied to the same CCTA images

FFR_AI_ predicts the FFR value based on the entire artery as opposed to only the stenotic segment. The model uses the cMPR images of the entire artery for the prediction. To enable direct comparison of the FFR_CT_ value to the FFR measurement distal to the stenosis of interest, the 3D FFR_CT_ view was used to match the stenotic sites on the CCTA and ICA, as previously reported (Fig. 1). [8]

#### FFR_AI_ postprocessing and analysis

All CCTA images were sent to an Aquarius Intuition 3D-workstation (V4.5, TeraRecon, Durham, NC), which automatically segmented each contrast-enhanced coronary artery. If required, the centerlines were corrected manually by the same experienced cardiac radiologist (JFP), who was blinded to the FFR and FFR_CT_ values. As for the validation set, nine cMPR images at 40° intervals around the centerline were acquired and uploaded to CorEx. For each of the nine images, the AI model predicted whether the FFR was ≤ 0.80 or > 0.80. The mean of the nine predictions was computed and used to classify each artery as having a predicted FFR ≤ 0.80 or > 0.80.

### Statistical analysis

The statistical analyses were performed using SPSS^®^ version 29.0 (IBM, Armonk, NY). Normally distributed continuous variables were expressed as mean ± standard deviation. For the FFR_CT_ and FFR_AI_ values, sensitivity, specificity, positive predictive value (PPV), and negative predictive values (NPV) were computed with their 95% confidence intervals (95%CIs), using the FFR value as the reference standard. Diagnostic accuracy was computed as the sum of true positives (TP) and true negatives (TN) over the sum of TP, TN, false positives, and false negatives. Diagnostic accuracy of the CCTA and AI methods was compared by applying the chi-square test.

Proportion of agreement was computed between FFR_AI_ and FFR, between FFR_CT_ and FFR and between FFR_AI_ and FFR_CT_. Values of *p* < 0.05 were taken to indicate statistically significant differences.

## Results

We retrospectively included 37 patients, 25 men and 12 women, with 39 intermediate-grade coronary stenoses. Mean age was 61 ± 9 years overall, 59 ± 9 years in the men, and 67 ± 8 years in the women. Patient selection flowchart and the main patient characteristics have been reported elsewhere [8]. Of the 39 stenoses, 23 were on left anterior descending arteries, 11 on right coronary arteries, and five on left circumflex arteries. No adverse events on ICA or contrast administration occurred.

The mean FFR value was 0.85 ± 0.10 (range, 0.60–0.99). Of the 39 stenoses, 11 (28%) had FFR values ≤ 0.80 (range, 0.60–0.79).

Both FFR_AI_ and FFR_CT_ were determined successfully for each stenosis. Table 1 shows the contingency table for each method. The stenosis that was a false negative by FFR_AI_ had an FFR value of 0.78. The FFR values for the two false negatives by FFR_CT_ were 0.79 and 0.74, respectively.

**Table 1:** Contingency tables for FFR prediction by FFR_AI_ and FFR_CT_

	**FFR**_AI_	
**FFR**	Positive (≤ 0.80)	Negative (> 0.80)
Positive (≤ 0.80)	10	5
Negative (> 0.80)	1	23
	**FFR**_**CT**_	
**FFR**	Positive (≤ 0.80)	Negative (> 0.80)
Positive (≤ 0.80)	9	7
Negative (> 0.80)	2	21

FFR_AI_: estimation of fractional flow reserve by deep learning analysis of coronary computed tomography angiography (CCTA) images; FFR: fractional flow reserve determined during invasive coronary angiography; FFR_CT_: estimation of fractional flow reserve by computational fluid dynamics analysis of CCTA images

Table 2 reports the sensitivity, specificity, PPV, NPV, and diagnostic accuracy for FFR_AI_ and FFR_CT_, using FFR as the reference standard. Accuracy in predicting FFR ≤ 0.80 was not significantly different between the two methods (*p* = 0.12). Proportion of agreement was 85% (33/39) between FFR_AI_ and FFR, 77% (30/39) between FFR_CT_ and FFR, and 77% (30/39) between FFR_AI_ and FFR_CT_.

**Table 2:** Diagnostic performance of FFR_AI_ and FFR_CT_ compared to fractional flow reserve measured during invasive coronary angiography

	FFR_AI_ (95%CI)	FFR_CT_ (95%CI)
Sensitivity	91 (57–99)%	82 (48–97)%
Specificity	82 (62–93)%	75 (55–89)%
PPV	67 (39– 87)%	56 (31–79)%
NPV	96 (77–99)%	91 (71–99)%
Accuracy	85 (64–96)%	77 (53–97)%

FFR_AI_: estimation of fractional flow reserve by artificial-intelligence deep learning analysis of coronary computed tomography angiography (CCTA) images; FFR_CT_: estimation of fractional flow reserve by computational fluid dynamics analysis of CCTA images; 95%CI: 95% confidence interval; PPV: positive predictive value; NPV: negative predictive value

## Discussion

In this proof-of-concept study, the AI DLM was as accurate as computational fluid dynamics analysis in predicting whether FFR was ≤ 0.80 or > 0.80 in intermediate-grade coronary artery stenoses. [10–12]

The 85% diagnostic accuracy of our model was higher than the 76% value obtained using a 3D, fully automatic, multiple-neural-network model based on 131 datasets. [7] Another AI approach using a support vector machine with 2D MPR images obtained from centerlines in 137 patients (192 coronary arteries) had 66–79% accuracy depending on whether prediction targeted FFR values ≤ 0.70 to ≥ 0.90.^13^ However, this study was not confined to arteries with intermediate-grade stenoses. Intermediate-grade stenoses are challenging, as only one third of these lesions are ischemic and require revascularization. [4] The inclusion of a broader range of stenosis severities may have increased the diagnostic accuracy of our AI model. A strength of our study is the large training set comprising 4500 labeled images from 500 stenotic arteries in 413 patients. The previously reported models used smaller training datasets, which may have influenced the diagnostic performance. [7, 13] Our model was trained on segmented cMPR images, with expert corrections, which may facilitate the learning efficiency compared to models using directly axial images. When designing AI models to perform medical tasks, great care is required given the multiple potential sources of bias, and extensive studies must then be performed to assess general applicability. [14, 15] AI models for FFR_AI_ prediction can be expected to improve over time as increasingly large training datasets are used.

Our study is the first to compare an AI model with FFR_CT_ in intermediate-grade coronary stenoses. FFR_CT_ was developed over a decade ago as a non-invasive method for estimating FFR. [16] A review of randomized controlled trials in patients with intermediate-grade stenoses showed that FFR_CT_ had high sensitivity and NPV for predicting FFR ≤ 0.80, similar to the values shown in our study. [17] One of the reviewed trials demonstrated that FFR_CT_ was nearly as sensitive and considerably more specific (79% vs. 34%) compared to morphological evaluation on CCTA images. [17, 18] In several previous studies with larger sample sizes than ours, the diagnostic performance characteristics of FFR_CT_ were in line with those found in our cohort. [11, 12, 16] Proportion of agreement was high between all methods and highest between FFR_AI_ and FFR. All three false negative cases, one for FFR_AI_ and two for FFR_CT_ were within or around the FFR ‘grey zone’ of 0.75–0.80. [19] For such values, the appropriateness of revascularization as opposed to medical treatment alone is difficult to assess, [20] as the optimal FFR cutoff for determining revascularization remains debated. [21, 22] The clinical benefits of revascularization are not expected to be as marked for lesions with borderline FFR values, compared to stenoses of greater severity. In predominantly single-vessel disease, FFR cutoffs of 0.72 to 0.75 have been suggested for predicting myocardial ischemia. [23]

FFR_CT_ determination has gained a place as a non-invasive tool for decreasing the number of unnecessary ICAs, thereby diminishing risks to patients and costs to healthcare systems.

AI for FFR prediction has several advantages over morphological CCTA analysis. AI models for automatically and accurately classifying stenoses as < 50% vs. ≥50% are available [5, 24, 25] and could be used for selecting those patients who require additional functional evaluation. Our model uses cMPR reconstructions for FFR prediction enabling instant FFR_AI_ evaluation on CCTA images into the daily clinical workflow.

The main limitations of our study are the retrospective data collection, single-center recruitment, and small sample size. However, we included only patients with intermediate-grade stenoses, that is, posing the greatest management challenges. The results of AI model requires confirmation in a multicenter prospective study with a larger sample size. Second, the model uses cMPR images with manual centerline adjustment when needed. The accuracy of centerline adjustment may vary across operators, notably with poor-quality images or heavily calcified stenoses. An AI model capable of performing automatic contouring and segmentation, would eliminate this drawback. Finally, our AI model uses a dichotomous outcome, predicting FFR as ≤ 0.80 or > 0.80, a continuous outcome would be more clinically relevant especially in borderline lesions. A global-artery evaluation by FFR_AI_ might help to assess extensive lesions but would require a dedicated AI model.

In conclusion, FFR_AI_ had similar diagnostic accuracy as FFR_CT_ for predicting intermediate-grade stenoses with FFR ≤ 0.80. This method may be an accurate non-invasive imaging modality for guiding the therapeutic management of patients with intermediate-grade stenoses. Further assessment of these preliminary findings in a larger, prospective population is required.

## Data Availability

No datasets were generated or analysed during the current study.

## References

[1] Knuuti J, Wijns W, Saraste A et al (2020) 2019 ESC guidelines for the diagnosis and management of chronic coronary syndromes. Eur Heart J 41:407–477 31504439 10.1093/eurheartj/ehz425

[2] Gulati M, Levy PD, Mukherjee D et al (2022) 2021 AHA/ACC/ASE/CHEST/SAEM/SCCT/SCMR Guideline for the evaluation and diagnosis of chest pain: a report of the American College of Cardiology/American Heart Association Joint Committee on Clinical Practice guidelines. J Cardiovasc Comput Tomogr 16:54–122 34955448 10.1016/j.jcct.2021.11.009

[3] Ghekiere O, Dewilde W, Bellekens M et al (2015) Diagnostic performance of quantitative coronary computed tomography angiography and quantitative coronary angiography to predict hemodynamic significance of intermediate-grade stenoses. Int J Cardiovasc Imaging 31:1651–1661 26323355 10.1007/s10554-015-0748-1

[4] Lawton JS, Tamis-Holland JE, Bangalore S et al (2022) 2021 ACC/AHA/SCAI Guideline for coronary artery revascularization: a report of the American College of Cardiology/American Heart Association Joint Committee on Clinical Practice guidelines. Circulation 145:E18–E114 34882435 10.1161/CIR.0000000000001038

[5] Paul JF, Rohnean A, Giroussens H et al (2022) Evaluation of a deep learning model on coronary CT angiography for automatic stenosis detection. Diagn Interv Imaging 103:316–323 35090845 10.1016/j.diii.2022.01.004

[6] Zreik M, Van Hamersvelt RW, Wolterink JM, Leiner T et al (2019) A recurrent CNN for automatic detection and classification of coronary artery plaque and stenosis in coronary CT angiography. IEEE Trans Med Imaging 38:1588–1598 30507498 10.1109/TMI.2018.2883807

[7] Kumamaru KK, Fujimoto S, Otsuka Y et al (2020) Diagnostic accuracy of 3D deep-learning-based fully automated estimation of patient-level minimum fractional flow reserve from coronary computed tomography angiography. Eur Heart J Cardiovasc Imaging 21:437–445 31230076 10.1093/ehjci/jez160

[8] Ghekiere O, Bielen J, Leipsic J et al (2019) Correlation of FFR-derived from CT and stress perfusion CMR with invasive FFR in intermediate-grade coronary artery stenosis. Int J Cardiovasc Imaging 35:559–568 30284138 10.1007/s10554-018-1464-4

[9] Neumann FJ, Sousa-Uva M, Ahlsson A et al (2019) 2018 ESC/EACTS guidelines on myocardial revascularization. Eur Heart J 40:87–165 30615155 10.1093/eurheartj/ehy855

[10] Tesche C, De Cecco CN, Albrecht MH et al (2017) Coronary CT angiography-derived fractional flow reserve. Radiology 285:17–33 28926310 10.1148/radiol.2017162641

[11] Kruk M, Wardziak Ł, Demkow M et al (2016) Workstation-based calculation of CTA-Based FFR for Intermediate Stenosis. JACC Cardiovasc Imaging 9:690–699 26897667 10.1016/j.jcmg.2015.09.019

[12] Nakazato R, Park HB, Berman DS et al (2013) Noninvasive fractional flow reserve derived from computed tomography angiography for coronary lesions of intermediate stenosis severity: results from the DeFACTO study. Circ Cardiovasc Imaging 6:881–889 24081777 10.1161/CIRCIMAGING.113.000297

[13] Zreik M, Van Hamersvelt RW, Khalili N et al (2020) Deep learning analysis of coronary arteries in cardiac CT angiography for detection of patients requiring invasive coronary angiography. IEEE Trans Med Imaging 39:1545–1557 31725371 10.1109/TMI.2019.2953054

[14] Futoma J, Simons M, Panch T et al (2020) The myth of generalisability in clinical research and machine learning in health care. Lancet Digit Health 2:e489 32864600 10.1016/S2589-7500(20)30186-2 PMC7444947

[15] Liao J, Huang L, Qu M et al (2022) Artificial intelligence in coronary ct angiography: current status and future prospects. Front Cardiovasc Med 9:896366 35783834 10.3389/fcvm.2022.896366 PMC9247240

[16] Min JK, Leipsic J, Pencina MJ et al (2012) Diagnostic accuracy of fractional flow reserve from anatomic CT angiography. JAMA 308:1237–1245 22922562 10.1001/2012.jama.11274 PMC4281479

[17] Raja J, Seitz MP, Yedlapati N, Khouzam RN (2021) Can computed fractional Flow Reserve Coronary CT Angiography (FFRCT) offer an Accurate Noninvasive comparison to invasive coronary angiography (ICA)? The noninvasive CATH. A Comprehensive Review. Curr Probl Cardiol. ;46 10.1016/j.cpcardiol.2020.100642 32624193

[18] Nørgaard BL, Leipsic J, Gaur S et al (2014) Diagnostic performance of noninvasive fractional flow reserve derived from coronary computed tomography angiography in suspected coronary artery disease: the NXT trial (analysis of coronary blood flow using ct angiography: next steps). J Am Coll Cardiol 63:1145–1155 24486266 10.1016/j.jacc.2013.11.043

[19] Kang DY, Ahn JM, Lee CH et al (2018) Deferred vs. performed revascularization for coronary stenosis with grey-zone fractional flow reserve values: data from the IRIS-FFR registry. Eur Heart J 39:1610–1619 29529177 10.1093/eurheartj/ehy079

[20] Johnson NP, Tóth GG, Lai D et al (2014) Prognostic value of fractional flow reserve: linking physiologic severity to clinical outcomes. J Am Coll Cardiol 64:1641–1654 25323250 10.1016/j.jacc.2014.07.973

[21] Zimmermann FM, Ferrara A, Johnson NP et al (2015) Deferral vs. performance of percutaneous coronary intervention of functionally non-significant coronary stenosis: 15-year follow-up of the DEFER trial. Eur Heart J 36:3182–3188 26400825 10.1093/eurheartj/ehv452

[22] De Bruyne B, Pijls NHJ, Kalesan B et al (2012) Fractional flow reserve-guided PCI versus medical therapy in stable coronary disease. New Engl J Med 367:991–1001 22924638 10.1056/NEJMoa1205361

[23] Berry C, Corcoran D, Hennigan B et al (2015) Fractional flow reserve-guided management in stable coronary disease and acute myocardial infarction: recent developments. Eur Heart J 36:3155–3164c 26038588 10.1093/eurheartj/ehv206 PMC4816759

[24] Lin A, Manral N, McElhinney P et al (2022) Deep learning-enabled coronary CT angiography for plaque and stenosis quantification and cardiac risk prediction: an international multicentre study. Lancet Digit Health 4:e256–e265 35337643 10.1016/S2589-7500(22)00022-X PMC9047317

[25] Choi AD, Marques H, Kumar V et al (2021) CT ​evaluation ​by ​artificial ​intelligence ​for ​atherosclerosis, stenosis and vascular ​morphology ​(CLARIFY): ​A ​multi-center, international study. J Cardiovasc Comput Tomogr 15:470–476 34127407 10.1016/j.jcct.2021.05.004

